# Amino acid-assisted effect on hydrate-based CO_2_ storage in porous media with brine†

**DOI:** 10.1039/d4ra00330f

**Published:** 2024-03-19

**Authors:** Amirun Nissa Rehman, Cornelius Borecho Bavoh, Mohd Yusuf Khan, Bhajan Lal

**Affiliations:** a Interdisciplinary Research Center for Hydrogen Technologies and Carbon Management, King Fahd University of Petroleum & Minerals Dhahran 31261 Saudi Arabia amirunrehman2728@gmail.com; b Department of Chemical and Petrochemical Engineering, University of Mines and Technology P.O. Box 237 Tarkwa Ghana; c Chemical Engineering Department, Universiti Teknologi PETRONAS Bandar Seri Iskandar 32610 Perak Darul Ridzuan Malaysia

## Abstract

CO_2_ storage as hydrates in porous media is a promising method for storing carbon dioxide (CO_2_). However, the sluggish formation kinetics of hydrates urge the need to focus on the use of additives (promoters) to accelerate hydrate kinetics. This study investigates the effect of amino acid solutions in brine on CO_2_ hydrate formation and dissociation kinetics in quartz sand particles QS-2 (0.6–0.8 mm) with 38% porosity. The amino acids l-methionine (l-meth), l-isoleucine (l-iso), and l-threonine (l-threo) were studied at 0.2 wt% using an autoclave hydrate reactor at 4 MPa and 274.15 K in the presence and absence of salt (3.3 wt% NaCl) in 100% water saturation. The hydrate dissociation kinetics was studied at a temperature of 277.15 K. These conditions represent the normal seabed temperature range in Malaysia and hence were used for testing CO_2_ hydrate formation and dissociation kinetics in quartz sand in this study. Further, CO_2_ hydrate formation and dissociation experiments were conducted with sodium dodecyl sulphate (SDS) and brine systems as standards for comparison. The findings reveal the best kinetics for l-meth exhibiting the highest CO_2_ hydrate storage capacity. l-meth recorded a gas-to-hydrate conversion ratio of about 93% at 0.2 wt% in quartz sand with brine. Moreover, l-meth exhibited the lowest hydrate dissociation rate compared to l-iso and l-threo systems, thereby enhancing CO_2_ hydrate stability in quartz sand. Comparatively, l-meth enhanced the storage capacity by 36% and reduced the induction time by more than 50% compared to conventional promoter SDS in quartz sand with brine, suggesting it to be favorable for CO_2_ storage applications. CO_2_ hydrate nucleation time was predicted in quartz sand with and without the best-studied amino acid l-meth system with high prediction accuracy and an absolute average deviation of 2.4 hours. The findings of this study substantiate the influence of amino acids in promoting the storage capacity of CO_2_ in sediments as hydrates.

## Abstract 2

CO_2_ storage as hydrates in porous media is a promising method for storing carbon dioxide (CO_2_).

## Graphical abstract

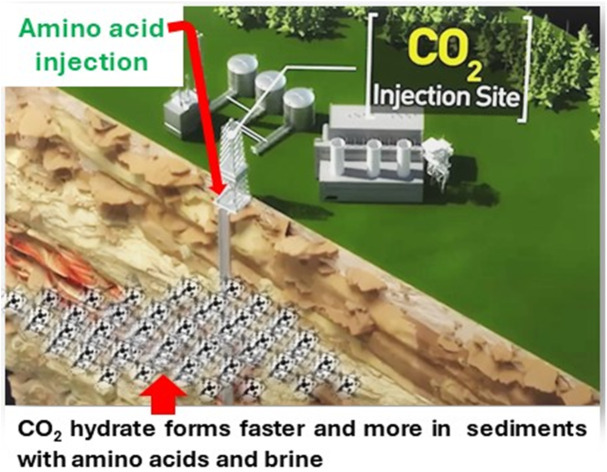

## Introduction

CO_2_ sequestration is an important component of the carbon capture, utilization, and storage (CCUS) value chain. It is one of the prime concerns around the globe, with several ongoing attempts to attain net zero CO_2_ emissions. This has been achieved through capturing CO_2_ from different sources. Several studies have investigated CO_2_ capture using different materials, such as porous polymers and metal–organic frameworks, attaining high CO_2_ capture capacity.^1–5^ Though conventional CO_2_ sequestration processes such as storing CO_2_ in depleted oil and gas reservoirs, saline aquifers, or utilizing CO_2_ in EOR applications are in practice, challenges facing storage capacity and ensuring long-term CO_2_ storage are critical factors that need to be addressed. In view of this, extensive research is underway to explore different techniques to permanently store CO_2_.

Geological CO_2_ storage is another approach that promises to store huge amounts of CO_2_ owing to the existence of large sequestration locations. On the contrary, CO_2_ disposal in sediments is challenged by leakage issues, where there is a high possibility of CO_2_ being released back into the atmosphere, thus causing further damage to the environment. In this regard, hydrate-based CO_2_ sequestration is an emerging technique with a high potential of storing large volumes of CO_2_ arising from its high volumetric capacity (1 m^3^ of hydrate can store about 120–180 m^3^ of gas at STP). Gas hydrates are solid inclusion compounds formed by the physical combination of gas molecules such as methane, ethane, propane, carbon dioxide, *etc.* and water molecules at favorable thermodynamic conditions (high pressure and low temperature).^6–9^ Herein, water molecules, being the host, form cage-like structures, trapping gas molecules as guests within the host cages, forming gas hydrate structure. Hydrate-based CO_2_ storage is advantageous because it is a solid form of CO_2_ storage that minimizes the chances of leakage unlike conventional CO_2_ storage techniques.

However, to store CO_2_ as hydrate in sediments, it is vital to select an appropriate location that could provide high CO_2_ hydrate storage capacity. This, in turn, is affected by several factors, particularly the sediment type and its properties based on the specific sequestration locations. Hydrate kinetics (formation and dissociation) is considerably affected by the properties of the sediment type, such as porosity, particle size, surface area, and pore size. Besides this, the sluggish kinetics of hydrate formation is another challenge that necessitates the use of additives (promoters) to enhance the hydrate formation process. Based on the choice of application, these additives could be thermodynamic or kinetic hydrate promoters. Kinetic hydrate promoters are also designated as low-dosage hydrate promoters (LDHP) due to the advantage that these can be used in low concentrations (<10 000 ppm). In practical industrial applications, usually lower concentrations are preferred, and hence, the need for low-dosage kinetic promoters arises. The role of kinetic promoters is to reduce the surface tension between the gas and liquid phase during hydrate formation. These promoters are in the form of surfactants or high-surface materials that interact with the water molecules during hydrate formation. Anionic surfactant sodium dodecyl sulphate (SDS) is the most widely studied kinetic promoter for CO_2_ hydrate formation. However, a typical CO_2_ hydrate storage operation discourages the use of additives, especially conventional unfriendly additives. Hence, there arises the need to search for new promoters that could replace the use of conventional promoters. In this regard, recent investigations have reported amino acids as potential gas hydrate promoters that are biodegradable and can enhance hydrate kinetics without any foam generation.^10^ It has also gained much application in other engineering fields, such as drilling.^11^

Amino acids as kinetic promoters for CO_2_ hydrate were first reported by Cai *et al.*^12^ They studied l-methionine at different concentrations and reported 0.2 wt% as the optimum concentration with enhanced CO_2_ uptake. Several other studies in the literature report the use of amino acids as kinetic promoters for CO_2_ as well as (CO_2_ + CH_4_) mixed gas systems.^13–20^ However, kinetic studies related to the use of amino acids for hydrate-based geological CO_2_ sequestration have been less focused in the literature. Pandey *et al.*^21^ studied the effect of l-valine, l-methionine, and l-histidine on CO_2_ hydrate formation kinetics in sediments and compared it with commercial promoter SDS. Considering induction time measurements, they concluded the kinetic promotion effect of l-histidine over l-methionine, l-valine, and SDS in pure water. Another study by Zhang *et al.*^22^ reports the kinetic promotion effect of l-methionine over d-leucine for CO_2_ hydrate using the initial stirring method to enhance hydrate formation kinetics. However, most of the literature studies using amino acids as promoters are conducted in pure water and do not represent the actual subsurface condition that contains brine. The behavior of amino acids could change in the presence of brine as opposed to pure water, which could affect the hydrate formation and stability kinetics. There is only a handful of studies that report CO_2_ hydrate formation kinetics with amino acids in porous sediments containing brine. In addition, the impact of amino acids on hydrate dissociation kinetics in sediments with brine is limited. It is critical to gain a deeper understanding of the effect of amino acids on CO_2_ hydrate formation and stability kinetics with brine in sediments. This knowledge will assist in selecting a potential location and a good promoter that can provide high CO_2_ storage capacity for the practical implementation of hydrate-based CO_2_ sequestration in sediments.

This study evaluates the effect of amino acids, l-threo, l-meth and l-iso, on CO_2_ hydrate formation and dissociation kinetics in quartz sand with brine. These amino acids were studied at 0.2 wt% in the presence of 3.3 wt% NaCl solution as brine. Further, CO_2_ hydrate formation and dissociation experiments were also conducted with base brine and 0.2 wt% SDS systems as standard for comparison. The findings from this study provide valuable insights on the kinetic promotion effect of amino acids in the presence of brine for CO_2_ storage as hydrates in sediments.

## Methodology

### Sample materials and procedure

**Fig. 1:**
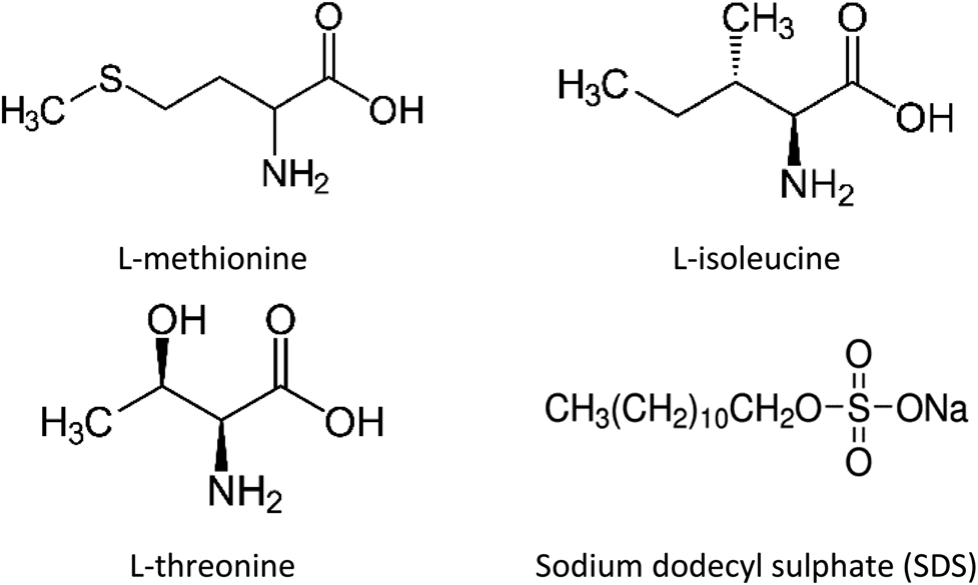
Chemical structures of amino acids and SDS.

In this study, unconsolidated quartz sand with particle sizes (0.6–0.8 mm) and porosity ∼38% (Fig. S1 ESI file†) was used to simulate the natural sediment system. The quartz sand was washed with deionized water and dried in an oven prior to its usage. The porosity of the quartz sand was determined using the volume saturation method.^23^ Table S1 (ESI file†) presents the data obtained from the BET analysis and Fig. S2 (ESI file†) shows the FESEM image of the quartz sand. The surface area of the quartz sand obtained from BET measurements was 1.1000 m^2^ g^−1^. The chemical structure and details of the gas hydrate kinetic promoters (amino acids and SDS) used in this work are shown in Fig. 1 and Table 1, respectively. All the chemicals were used without further purification or processing. The amino acid solutions were prepared in the brine system (3.3 wt% NaCl) and were studied at 0.2 wt% concentration. This was done to understand the effect of amino acids in the brine system on CO_2_ hydrate kinetics in the presence of quartz sand.

**Table 1:** List of chemicals

Chemicals	MW (gmol^−1^)	Purity (%)	Supplier
Deionized water			
Carbon dioxide	44.01	99.99	Gas Walker
Sodium chloride (NaCl)	58.44	99.99	Sigma-Aldrich
Unconsolidated quartz sand	60.08	Malaysian sea sand	Perak
l-Methionine	149.21	98	ACROS ORGANICS
l-Isoleucine	131.17	99	ACROS ORGANICS
l-Threonine	119.12	98	ACROS ORGANICS
Sodium dodecyl sulphate	288.38	99	Sigma-Aldrich

### Experimental setup

**Fig. 2:**
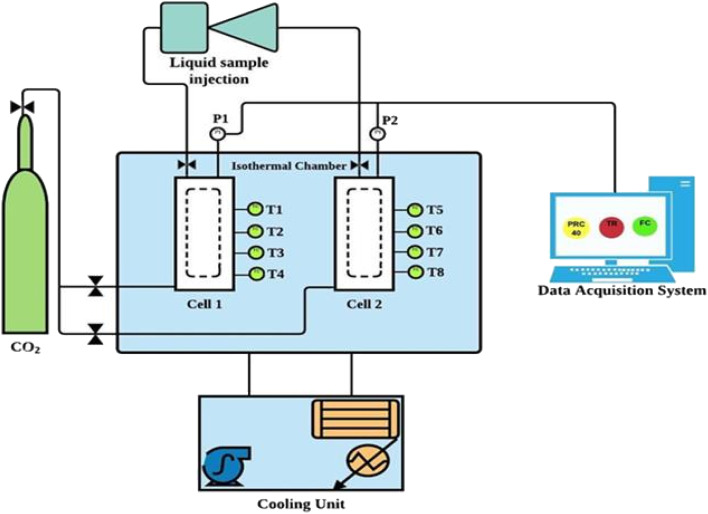
Schematic diagram of the sandstone hydrate reactor.^7^

Fig. 2 illustrates the schematic diagram of the experimental setup used in this study to investigate CO_2_ hydrate formation and dissociation kinetics in quartz sand with amino acids in brine system. More details on the experimental setup can be found in our previous studies.^7,24^ In brief, the setup consists of two stainless steel reactor cells, each having a volume capacity of about ∼700 ml that can withstand a maximum pressure of 150 bar. There are four Pt100 temperature sensors (T1, T2, T3 and T4) installed at the side of each reactor cell (Fig. S3 ESI file†). The temperature of all four temperature sensors varied by 0.3 K depending on the location of the sensors within the reactor. Further, two GP-M250 Keyence Japan pressure sensors are installed, one at the top and the other at the bottom of the reactor cell. These sensors monitor the variation in pressure and temperature inside the reactor with an accuracy of ±0.05 MPa and ± 0.05 K, respectively, during the experiment. Both the reactor cells are placed inside a water bath, as illustrated in Fig. 2. An external chiller connected to the water bath controls the water bath temperature.

The water bath containing glycol and water mixture circulates and monitors the system temperature during the hydrate testing experiment. The experimental setup is connected to the data acquisition system that records the temperature and pressure changes every 60 s during the entire experiment.

### Experimental procedure

#### Hydrate formation procedure

**Fig. 3:**
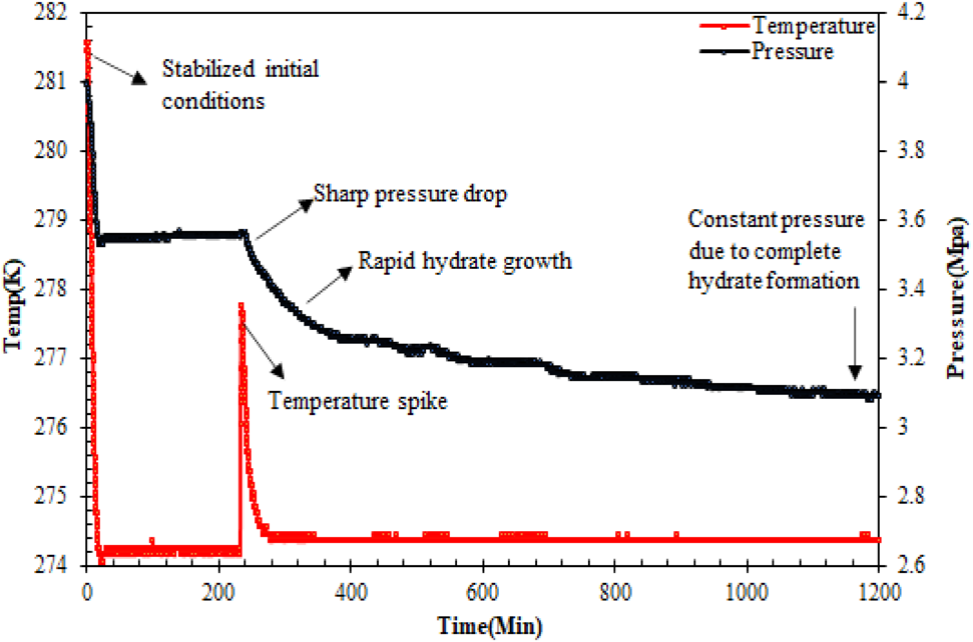
Typical pressure/temperature *vs.* time curve during CO_2_ hydrate formation in quartz sand.^19^

For the CO_2_ hydrate testing experiment in the sandstone hydrate reactor, initially, 420 g of unconsolidated quartz sand was used to prepare the sand bed, achieving a bed height of 6.5 cm. For more details on the sand bed preparation, readers may refer to our earlier studies.^7,24,25^ In brief, the sand bed was prepared in several stages with the careful addition of quartz sand and water/brine (3.3 wt% NaCl)/amino acid solutions in brine alternately, avoiding the formation of air pockets. The water saturation was maintained at 100% to mimic practical seabed conditions for hydrate-based geological CO_2_ sequestration. Since it is the same type of sand, the amino acid solutions in brine have a loading capacity of 140 ml. Subsequently, both the reactor cells were placed inside the water bath that contained the water–glycol mixture. Prior to the start of the experiment, the reactor cells were vacuumed with CO_2_ gas, ensuring the removal of excess air from the system. The system was then maintained at its initial pressure and temperature of 4 MPa and 282.15 K, respectively. After the system stabilized at the initial operating conditions for over 24 hours with no leakage, the CO_2_ hydrate formation experiment was started by reducing the system temperature to the experimental temperature of 274.15 K. Hydrate formation was noticed by a sudden drop in system pressure and simultaneous increase in system temperature as shown in Fig. 3. The stable pressure and temperature in the system for over three weeks indicated the completion of the hydrate formation experiment. It is assumed that there is no hydrate formation taking place in the reactor after 3 weeks of constant pressure. Subsequent runs were performed similarly with different amino acid solutions in the brine system in quartz sand.

### Hydrate formation kinetic evaluation indicators

#### CO_2_ hydrate nucleation time

The time for hydrate formation is estimated by calculating the induction time, which is defined as the time spent from the start of the experiment until the appearance of the first observable/measurable volume of hydrate crystal formed. In this work, the CO_2_ hydrate induction time was evaluated using eqn (1), as depicted in Fig. 3.

1 *t*_i_ = *t*_s_ − *t*_h_

where *t*_s_ represents the time taken by the system to stabilize at the initial operating conditions, and *t*_h_ is the time spent to observe the initiation of hydrate formation within the system.

#### Moles of CO_2_ uptake

Calculating the difference in moles of gas present initially (at time *t* = 0) and after time *t* of the experiment in the reactor yields the moles of CO_2_ consumed during the hydrate formation experiment. This is evaluated using experimental pressure and temperature using the real gas equation, where Δ*n*_H_ represents the moles of CO_2_ gas consumed, as shown in eqn (2).^26^2
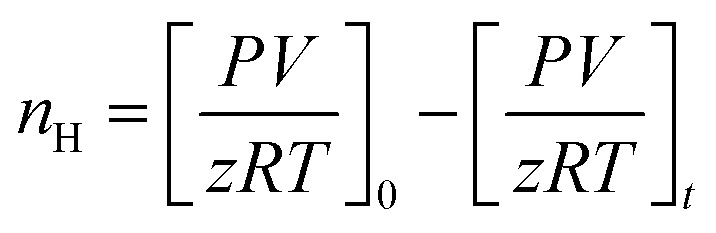
where *P* is the system pressure, *V* is the CO_2_ phase volume, *T* is the system temperature, and *R* represents the universal gas constant. The compressibility factor *z* is calculated using Pitzer's correlations.^27^

#### Initial CO_2_ hydrate formation rate

In this study, the CO_2_ hydrate formation rate was calculated using eqn (3) below.^7^3
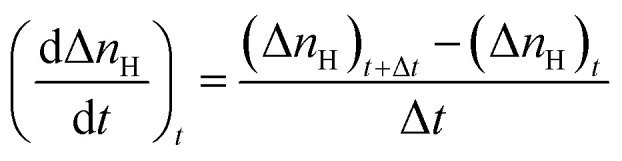


#### CO_2_ to hydrate conversion

During hydrate formation, CO_2_ storage capacity is quantified by estimating the gas (CO_2_) to hydrate conversion (*C*_gh_) ratio. A higher conversion ratio denotes higher CO_2_ storage capacity in sediments. It is calculated using eqn (4) below.^28^4
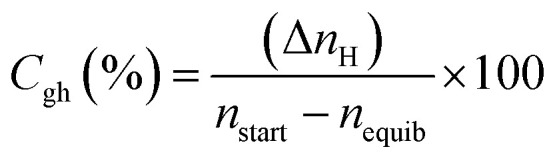
where (Δ*n*_H_) shows the moles of CO_2_ gas consumed (eqn (2)), *n*_start_ represents the amount of CO_2_ (gas phase) in the system before the start of the experiment and *n*_equib_ is the remaining amount of CO_2_ (gas phase) in the reactor upon complete hydrate formation.

#### H_2_O to hydrate conversion

The sediment properties regulate the water-to-hydrate conversion ratio. In this study, the water-to-hydrate conversion ratio (*C*_wh_) was estimated by employing eqn (5).5
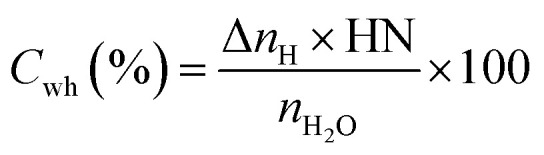
where Δ*n*_H_ and *n*_H_2_O_ are the moles of CO_2_ gas taken for hydrate formation (eqn (2)) and the total moles of initial water injected in the hydrate cell.^28,29^ HN is the hydration number for CO_2_ hydrates. (HN = 5.75 in this study).^28^

### Hydrate dissociation procedure

**Fig. 4:**
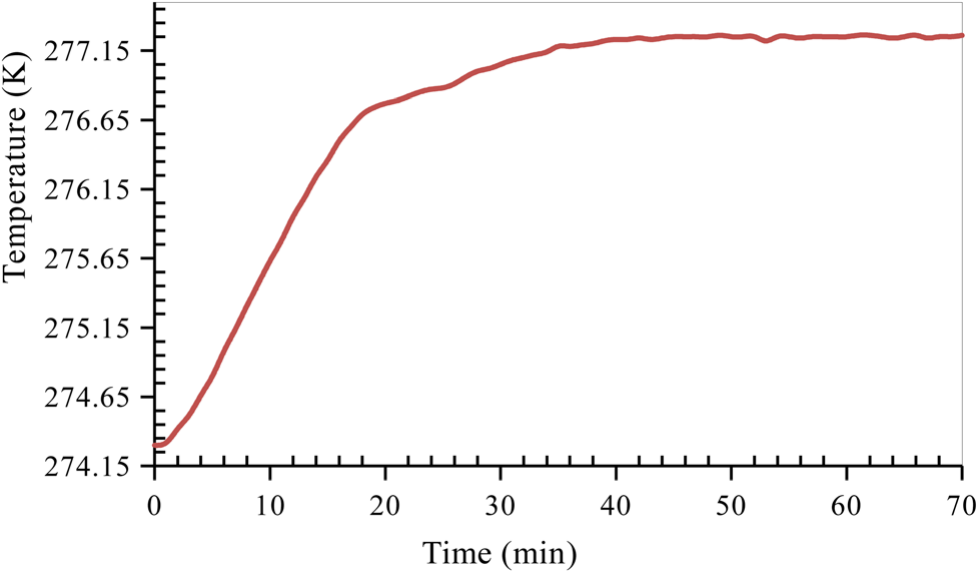
Temperature *versus* time curve of CO_2_ hydrate dissociation in quartz sand.

After the completion of the hydrate formation experiment, the CO_2_ hydrate dissociation experiment is carried out. Before starting the hydrate dissociation experiment, the reactor cell is stabilized by releasing excess pressure from the reactor cell and bringing it to a pressure close to approximately 10–15% of the equilibrium pressure at the corresponding experimental temperature. This was done to make sure there was no excess free gas in the reactor so that hydrate dissociation could be measured accurately. Because of the absence of a stirring mechanism in the sandstone hydrate reactor, it is essential to allow sufficient time for the system to stabilize. Once the system had stabilized, the formed CO_2_ hydrates were dissociated by heating the system to the experimental dissociation temperature of 277.15 K (Fig. 4). As the hydrate dissociation experiment proceeds, the reactor pressure increases slowly due to the dissociation of formed CO_2_ hydrates. The system is at experimental temperature (277.15 K) and pressure (10–15% below 1.7 MPa) for about 24 h until no further variation in pressure is observed. This indicates the completion of the dissociation experiment. The temperature and pressure changes within the reactor are monitored and recorded every 60 s throughout the experiment.

### Hydrate dissociation kinetic evaluation indicators

#### Moles of CO_2_ recovered

The estimation of the CO_2_ gas recovered at any given time relies on determining the moles of CO_2_ gas recovered (Δ*n*_cr_) from the hydrate dissociation experiment. This was calculated by subtracting the number of moles of CO_2_ gas released at time *t* from the initial number of moles of CO_2_ gas present at the start of the experiment (*t* = 0) in the reactor, as shown in the equation below6
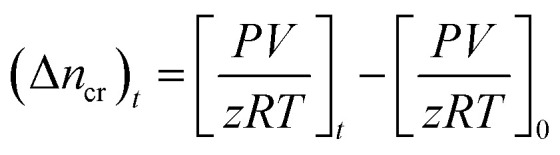


The normalized CO_2_ recovery curves were estimated by eqn (7) below,7
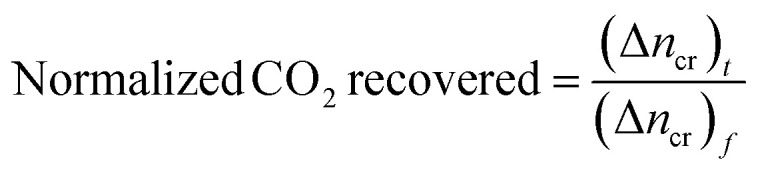
where (Δ*n*_cr_)_*t*_ indicates the total moles of CO_2_ gas recovered at the completion of the hydrate dissociation experiment. The time required to achieve 90% CO_2_ recovery is estimated as the equivalent time taken to achieve 90% normalized CO_2_ recovered from the plot of CO_2_ recovered against time.

#### CO_2_ hydrate dissociation rate

In this study, the CO_2_ hydrate dissociation rate was evaluated using the following equation^30^8
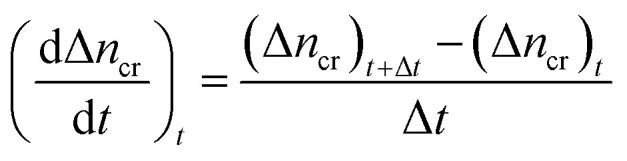


### CO_2_ hydrate nucleation prediction in quartz sand

#### Prediction model details

In this study, CO_2_ hydrate nucleation prediction was done using Classical Nucleation Theory, which is best known for predicting hydrate induction time. The generalized and simplified form of the CNT in describing and estimating the CO_2_ hydrate nucleation time is shown in eqn (9)9
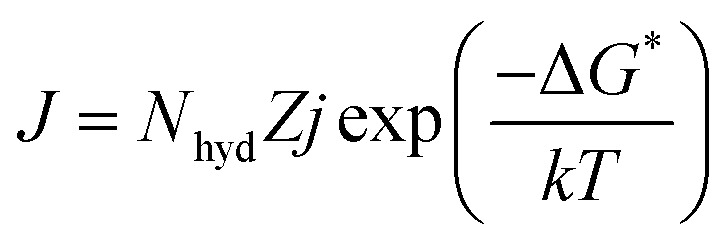
where *k* denotes the Boltzmann constant, and −ΔG* represents the free energy needed to form a hydrate critical nucleus size. The system temperature is *T*. *Z* is the likelihood that the hydrate critical nucleus size will yield a phase consisting of hydrates, *N*_hyd_ represents the amount of hydrate nucleation sites, and *j* is the rate at which the gas molecules stick to form the hydrate nucleus.

Considering that a spherical cap model hydrate embryo-like is formed, eqn (10) can be used to estimate the free energy required to attain a hydrate critical nucleus size.10
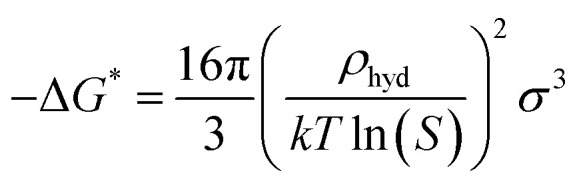
where *σ* denotes the CO_2_ hydrate surface tension,^31^*S* represents the supersaturation ratio, *T* is the system temperature, and *ρ*_hyd_ represents CO_2_ hydrate density.^31^ Hence the rate of hydrate nucleation of CO_2_ system in this work can be estimated by simply substituting eqn (10) into (9).

## Results and discussion

In this work, the effect of amino acids (l-threo, l-meth and l-iso) on CO_2_ hydrate formation and dissociation kinetics in quartz sand with brine is studied. The amino acids were investigated at 0.2 wt% using a sandstone hydrate reactor at 4 MPa and 274.15 K in quartz sand. CO_2_ hydrate testing was further conducted using 0.2 wt% SDS and brine system (3.3 wt% NaCl) as a base for comparison to evaluate the performance of the amino acids on CO_2_ hydrate kinetics in quartz sand. Each CO_2_ hydrate formation and dissociation experiment in quartz sand was repeated twice for reproducibility, and their average values were reported. The details of the values are shown in Table S2 in the ESI file.† In addition, CO_2_ hydrate nucleation time was predicted in quartz sand with and without amino acids using classical nucleation theory. The findings from this work provide meaningful insights on the use of amino acids as promoters for hydrate-based geological CO_2_ sequestration.

### CO_2_ hydrate formation kinetics in quartz sand

#### Effect of amino acid solutions in brine on CO_2_ hydrate formation kinetics in quartz sand

CO_2_ hydrate formation kinetics was studied using 0.2 wt% of l-meth, l-iso, and l-threo solutions in 3.3 wt% brine at 4 MPa and 274.15 K in quartz sand (Table 2). Herein, the results are mainly discussed based on the hydrate nucleation time or induction time measurement, CO_2_ uptake, initial rate of hydrate formation, water-to-hydrate, and CO_2_-to-hydrate conversion ratios, as these are some of the key indicators during the hydrate formation process. Induction time refers to the time observed between the start of the experiment and the time when the first noticeable hydrate crystals form in the system. It is an important kinetic parameter that facilitates the understanding of when or after how long hydrates form in the system. Based on the magnitude of induction time, the system might exhibit promotion (short induction time) or inhibition (prolonged induction time). For hydrate-based CO_2_ sequestration in sediments, a lower induction time is preferred to form hydrates within a short period. Another vital kinetic parameter is the CO_2_ gas uptake that provides the CO_2_ storage capacity. To store a large volume of CO_2_ as hydrate, a higher CO_2_ uptake is desired, along with high water and gas-to-hydrate conversion ratios. Hydrate formation rate provides a measure of the rate at which hydrate nucleation and growth occurs. Because of the variable amount of hydrates formed in each run, the first 3 weeks of each hydrate formation experiment are considered for effective comparison of results.

**Table 2:** Measured CO_2_ hydrate formation kinetic data at 0.2 wt% of l-meth, l-iso, l-threo, and SDS in 3.3 wt% brine solution at 4 MPa and 274.15 K in quartz sand

System	Induction time (h)	CO_2_ gas consumed (mol)	CO_2_ gas uptake (mol mmol^−1^)	Hydrate formation rate (mmol h^−1^)	*C*_gh_ (%)	*C*_wh_ (%)
l-Methionine	100	0.6568	90.89	2.173	93.29	53.99
l-Isoleucine	112	0.2623	36.30	0.376	38.27	21.56
l-Threonine	16.6	0.4587	56.41	5.427	70.54	33.84
SDS	150	0.5349	66.69	1.422	80.75	40.01
Brine	28	0.4270	57.11	7.494	68.38	34.26

**Fig. 5:**
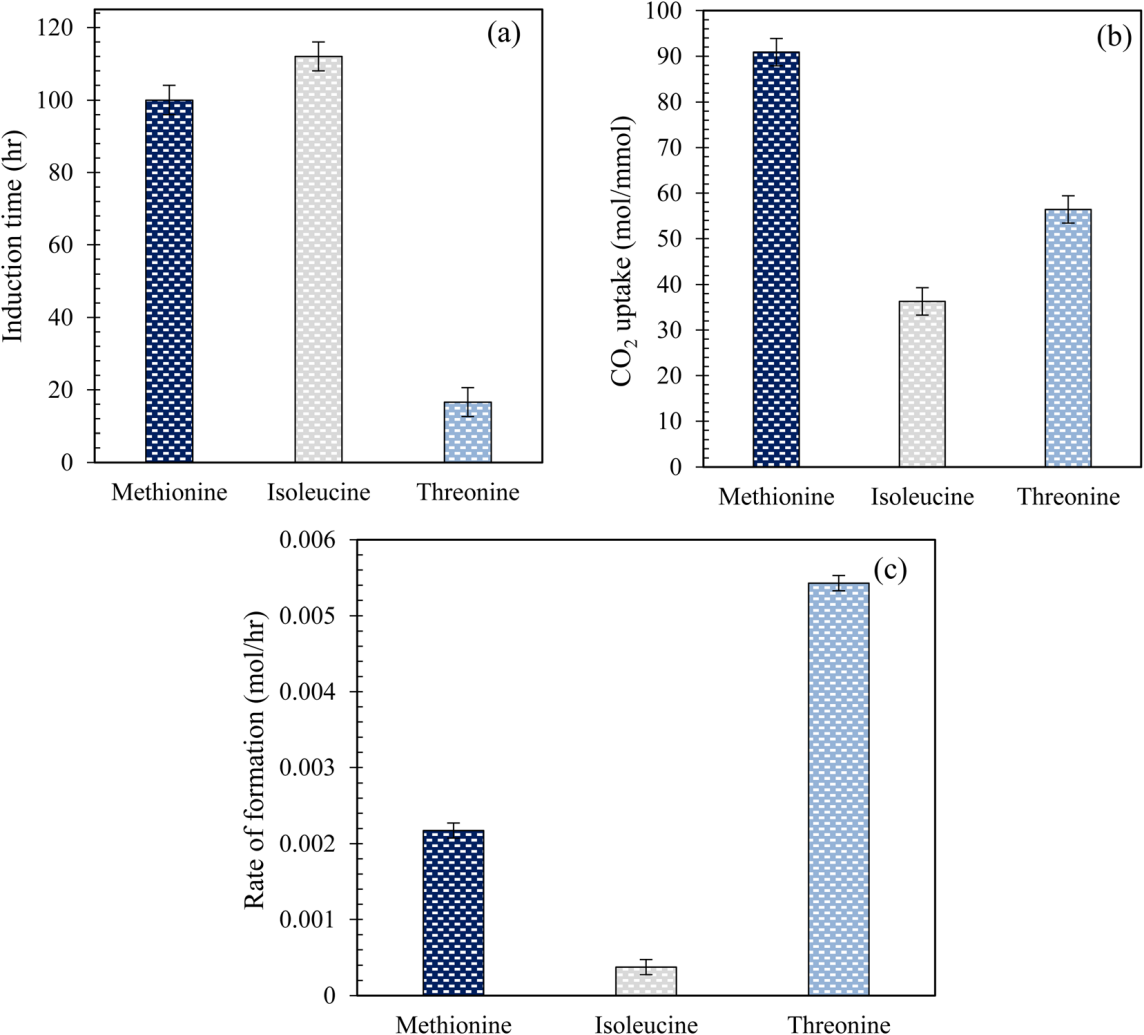
(a) Induction time; (b) CO_2_ uptake measurements; (c) rate (initial) of CO_2_ hydrate formation using 0.2 wt% of l-meth, l-iso, and l-threo in quartz sand with brine solution at 274.15 K and 4 MPa.

Fig. 5 illustrates the measured induction time and CO_2_ uptake during hydrate formation in quartz sand using l-threo, l-meth, and l-iso at 0.2 wt% in the brine system. As observed from Fig. 5(a), there is a considerable difference in the measured induction times of the studied amino acid systems in quartz sand. The induction time shown is the lowest for l-threo while the highest for l-iso, exhibiting a reduced kinetic promotion effect of l-iso while significant kinetic promotion effect for l-threo system. The induction time is higher by 12% in l-iso than in l-meth, indicating a slight promotion effect of l-meth over l-iso. However, the induction time is reduced by 83% in l-threo, as compared to l-meth, exhibiting a strong kinetic promotion effect of l-threo over l-meth. On the other hand, based on the CO_2_ uptake (Fig. 5(b)) measurements, l-meth exhibits optimum performance among the studied amino acid systems with the highest moles of CO_2_ uptake. This indicates a strong kinetic promotion effect of l-meth over l-threo and l-iso systems in quartz sand. CO_2_ uptake is reduced by 60% and 38% in l-iso and l-threo systems, respectively, as compared to l-meth exhibiting considerable inhibition effect of l-iso and l-threo on CO_2_ hydrate formation kinetics. The higher degree of kinetic inhibition for l-iso could be attributed to its low salt tolerance, which affects its solubility in brine. On the contrary, the low hydrophobicity of l-threo might be the cause for the lower degree of inhibition compared to l-iso, thus exhibiting high CO_2_ uptake during hydrate formation in quartz sand. Similar findings in the literature report higher gas uptake using l-meth while lower gas uptake using l-threo in pure water system.^16^ However, these findings were based on CO_2_ gas uptake measurements using aqueous solutions of amino acids without porous media.

Herein, the enhanced promotion effect of l-meth could be due to the synergistic effect of the hydrophilic amino group and carboxyl group along with favourable alkyl chain length that results in kinetic promotion during hydrate formation. Alkyl chain length is an important parameter that governs the kinetic promotion effect in case of hydrophobic amino acids, where very long or very short alkyl chain length might result in hydrate inhibition kinetics.^12^ In addition, the high solubility of l-meth in brine (salting-in effect) causes it to interact better with the hydroxyl groups on the quartz sand surface *via* covalent bonding as compared to l-iso and l-threo solutions in brine.^32^ This confirms that in case of l-meth, the maximum amount of CO_2_ gas and water is converted to hydrate as opposed to l-iso and l-threo systems in brine. Thus, l-meth adsorption on the quartz sand's surface increases the surface hydrophobicity of the quartz sand, promoting hydrate growth and causing more hydrates to form, resulting in high CO_2_ storage capacity.^33^

Fig. 5(c) shows the initial rate of CO_2_ hydrate formation using 0.2 wt% solutions of l-meth, l-iso, and l-threo in 3.3 wt% brine in quartz sand. As observed from Fig. 5(c), based on initial hydrate formation rate measurements, l-iso indicates significant inhibition with the lowest initial hydrate formation rate for CO_2_ while l-threo exhibits considerable promotion exhibiting the highest formation rate. The rate of hydrate formation is reduced by 82% in l-iso while increasing by more than twice in l-threo as opposed to l-meth, respectively. The highest formation rate in l-threo could be because of the polar nature of the side chain, facilitating its interaction with the water molecules resulting in an increased hydrate formation rate. Similar conclusions of high and low rates were reported using 0.5 wt% aqueous solutions of l-meth and l-threo, respectively.^16^ These findings were based on the gas uptake rate measured in pure water without porous media. On the other hand, Sa *et al.*^34^ reported an increase in the growth rate using l-isoleucine, which contradicts the findings in this study. However, their results were based on aqueous solutions of amino acids in the bulk system without any porous media, which could be the reason for the difference in the results.

**Fig. 6:**
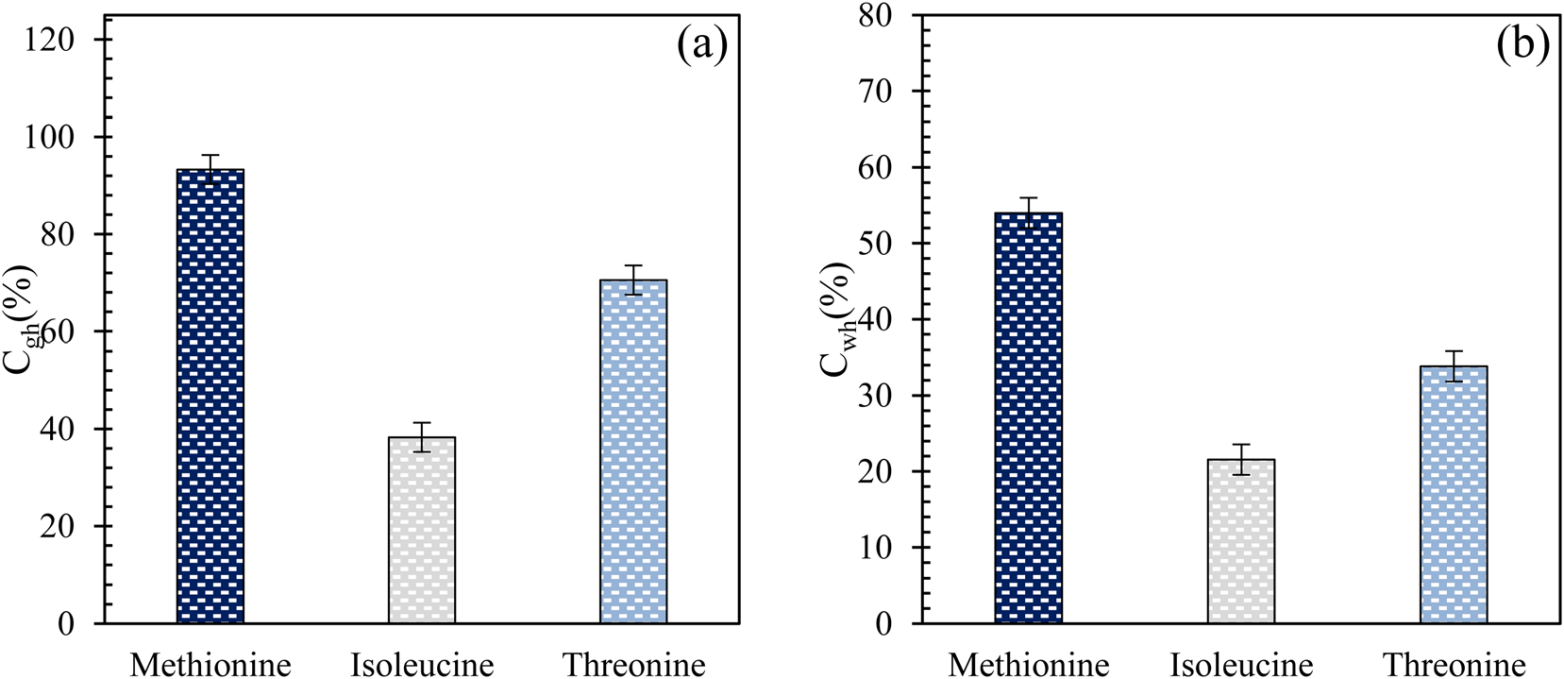
(a) Gas-to-hydrate and (b) water-to-hydrate conversion ratios (%) of l-meth, l-iso, and l-threo at 0.2 wt% in quartz sand with brine solution at 4 MPa and 274.15 K.

Fig. 6(a) and (b) present the CO_2_-to-hydrate and water-to-hydrate conversion ratios using 0.2 wt% l-meth, l-iso, and l-threo in brine system in quartz sand, respectively. The plots for both CO_2_-to-hydrate and water-to-hydrate conversion ratios follow a similar trend as CO_2_ gas uptake in this study. In both cases, the highest conversion ratio is achieved by l-meth followed by l-threo and l-iso systems in quartz sand. l-meth exceeds CO_2_-to-hydrate conversion ratio by 25% and 59% more than l-threo and l-iso systems in quartz sand, respectively. Similarly, water-to-hydrate conversion in l-meth exceeds by 37% and 60%, as opposed to l-threo and l-iso systems, respectively. This indicates the kinetic promotion effect of l-meth over l-iso and l-threo systems in quartz sand. It is speculated that the kinetic promotion effect of l-meth arises from the synergic interaction of the amino group (hydrophilic) and the carboxyl group along with the quartz sand's surface activity, favouring more hydrate formation. In addition, the surface hydroxyl groups of the quartz sand serve as the major adsorption sites, thereby providing nucleation sites for increased hydrate formation.^33^

For hydrate-based CO_2_ sequestration applications in marine sediments, one of the key parameters is the CO_2_ storage capacity, which is influenced by the moles of CO_2_ uptake. In this study, based on CO_2_ uptake measurements, l-meth exhibits the best performance in quartz sand with a brine system achieving the highest CO_2_ uptake, followed by l-threo. Hence, l-meth is further compared with commercial promoter SDS at 0.2 wt% to estimate the performance of each in quartz sand in the presence of brine system. In addition, both the systems were compared with the brine system as blank (without promoter) for effective comparison of results.

#### Comparison of induction time, CO_2_ uptake and formation rate for CO_2_ hydrate using l-meth, SDS and brine in quartz sand system

**Fig. 7:**
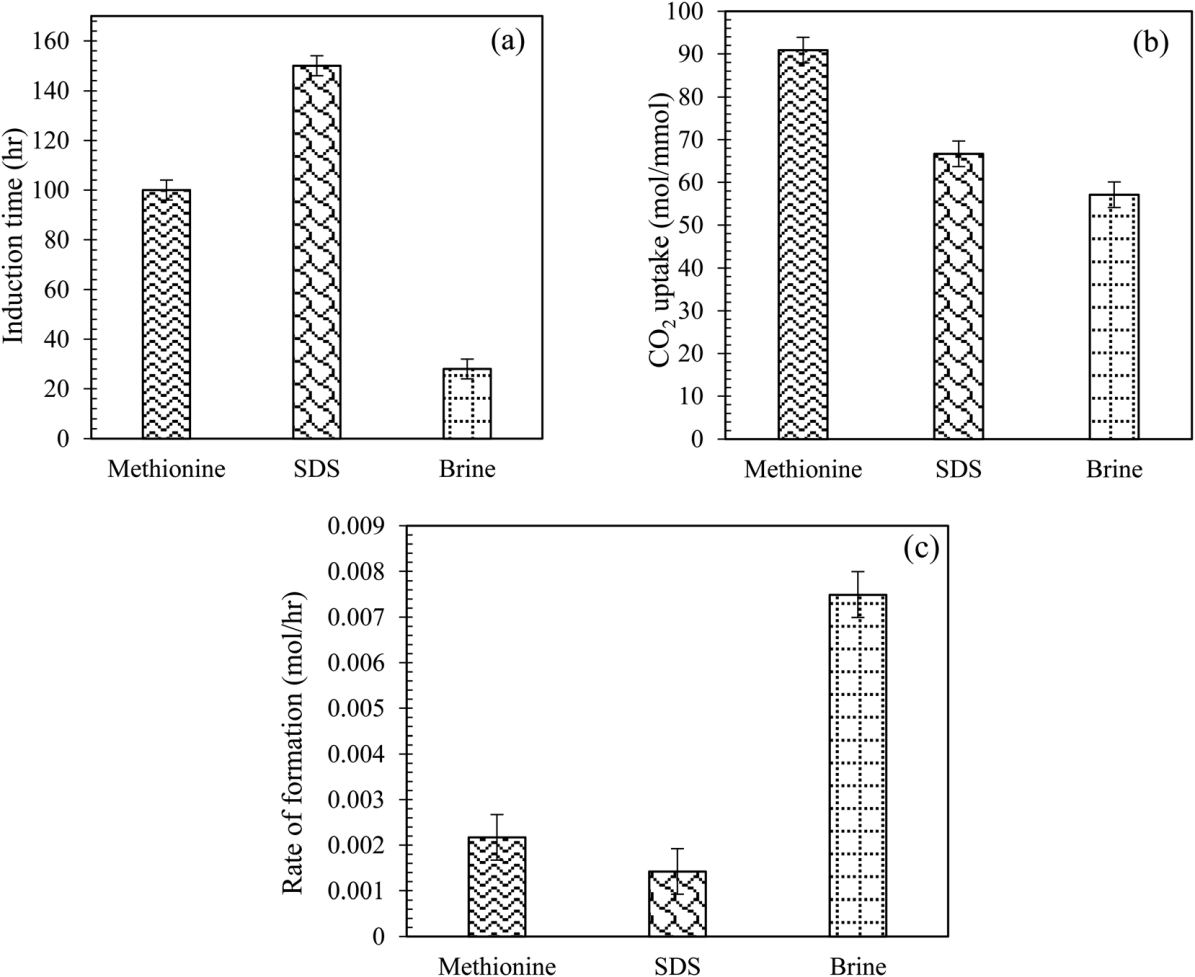
(a) Induction time, (b) CO_2_ uptake in moles, and (c) initial rate of CO_2_ hydrate formation using l-meth and SDS at 0.2 wt% in quartz sand with brine solution at 4 MPa and 274.15 K.

Fig. 7(a) and (b) illustrate the comparison of CO_2_ uptake and induction time measurements for CO_2_ hydrate formation using 0.2 wt% l-meth and SDS solution in the brine system (Table 2). Compared to the base system brine, l-meth exhibits better performance than SDS, considering both induction time and CO_2_ uptake measurements during hydrate formation in quartz sand. The induction time is reduced by 50% while CO_2_ uptake is increased by 26% in l-meth compared to SDS, respectively. This indicates the kinetic promotion effect of l-meth on CO_2_ hydrate formation kinetics in quartz sand with brine. This means the amount of CO_2_ gas and water forming hydrates was higher for the l-meth system, resulting in a high CO_2_ storage capacity. The kinetic promotion effect of l-meth could be attributed to its salting-in effect in the presence of brine, leading to high CO_2_ storage capacity in quartz sand. Thus, l-meth could possibly replace SDS for CO_2_ hydrate storage applications. Similar findings report promotion kinetics for methane hydrate using l-meth as opposed to SDS in quartz sand as porous media.^35^

Further, the rate of hydrate formation was compared for both l-meth and SDS solution with the brine system in quartz sand (Fig. 7(c)). As observed from Fig. 7(c), the initial rate of CO_2_ hydrate formation is the highest for base system brine. Among the other two systems, l-meth shows an increased hydrate formation rate than SDS, indicating its kinetic promotion effect for CO_2_ hydrate formation in quartz sand with brine. Thus, based on these findings, SDS could be successfully replaced with l-meth for CO_2_ storage applications in quartz sand with brine. However, Kang *et al.*^36^ reported an increased initial hydrate formation rate using SDS in silica gel based on driving force measurements in pure water.

#### CO_2_ hydrate nucleation time prediction

**Fig. 8:**
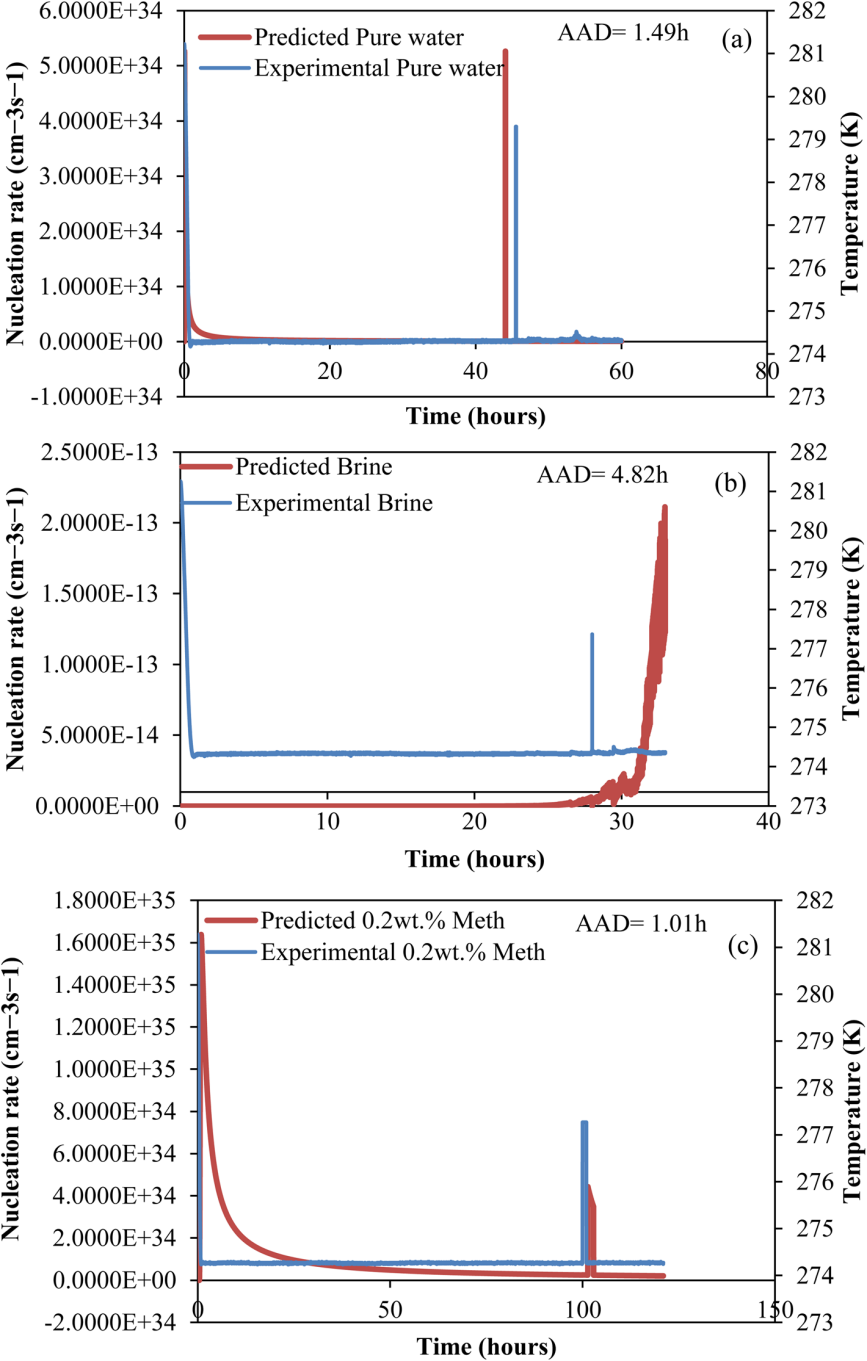
Predicted CO_2_ hydrate nucleation rate (*J*) and experiment temperature against time for all systems. (a) Pure water; (b) brine (base); (c) l-meth.

**Fig. 9:**
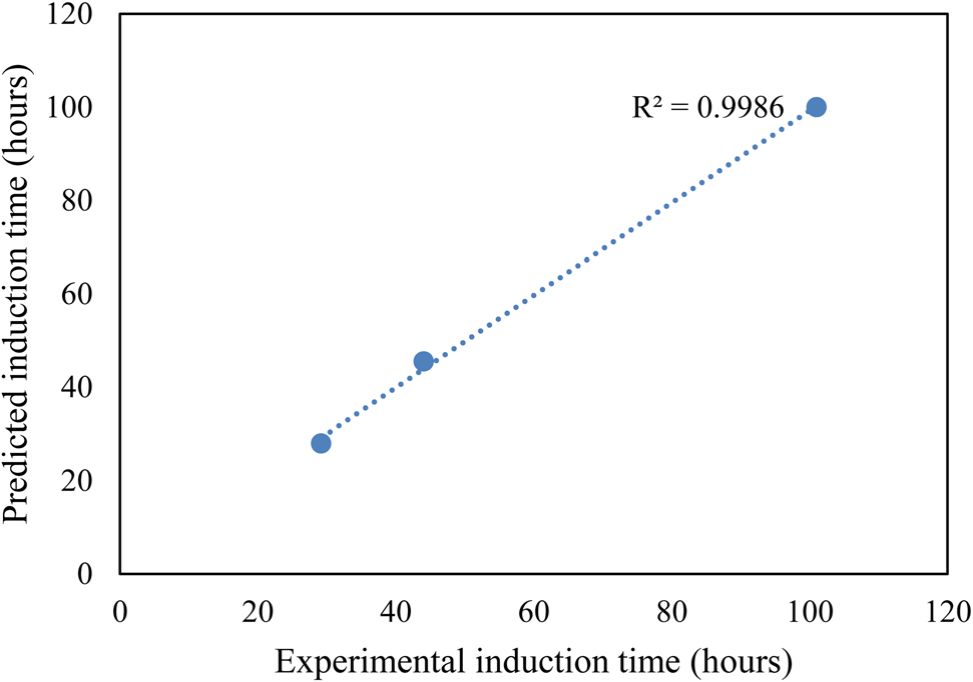
Experimental and CNT predicted induction time.

In this study, to effectively predict the CO_2_ hydrate formation induction time, the classical nucleation theory (CNT) model was used. Priority was given to the induction prediction because it is the foremost indicator of hydrate formation when storing CO_2_ as hydrates in sediments. The assumptions governing the CNT model consider that the system pressure at equilibrium controls the solvent concentration. Due to negligible temperature and temperature changes in the system, the hydrate formation process was assumed to obey the first-order equation at a constant cooling rate. The use of a homogenous CNT model is validated with the assumption that the hydrate formation cell walls are hydrophobic and very smooth. The CNT model predicted the CO_2_ hydrate nucleation onset time and the experimental CO_2_ hydrate formation temperature profile as a function of time, as presented in Fig. 8. In Fig. 8, the predicted CO_2_ hydrate induction time for all the systems agrees with the experimental data. The absolute average deviation prediction error was 2.4 hours with an adjusted *R*^2^ value of 0.9986 at a 95% confidence level (Fig. 9). This confirms that the CNT model can be effectively implemented for CO_2_ hydrate nucleation time prediction in quartz sand.

#### CO_2_ hydrate dissociation kinetics in quartz sand

Hydrate dissociation is an important factor concerning the stability of the formed hydrates, which ultimately influences the CO_2_ storage capacity as hydrates in sediments. To achieve a high CO_2_ storage capacity, a lower degree of hydrate dissociation is desired so that the formed hydrates can be stored stably for an extended period. In this study, CO_2_ hydrate dissociation was studied using 0.2 wt% l-meth, l-iso, and l-threo solutions in 3.3 wt% brine system using the sandstone hydrate reactor at 277.15 K (Table 3). This section discusses the hydrate dissociation kinetics based on the CO_2_ hydrate dissociation rate and time taken to release 90% of CO_2_ gas. Since the amount of hydrates formed in each experimental run was different, the amount of gas recovered was normalized against the total moles of CO_2._

**Table 3:** Measured CO_2_ hydrate dissociation data of 0.2 wt% of l-meth, l-iso, l-threo, and SDS in 3.3 wt% brine at 277.15 K in quartz sand

System	CO_2_ released (mol)	Rate of dissociation (mol h^−1^)	Time of 90% CO_2_ released (h)
l-Methionine	0.1408	0.1870	2.75
l-Isoleucine	0.1781	0.2154	3.18
l-Threonine	0.1321	0.2029	1.6
SDS	0.1335	0.1383	2.63
Brine	0.1266	0.2243	1.07

#### Effect of amino acid solutions in brine on CO_2_ hydrate dissociation kinetics in quartz sand

**Fig. 10:**
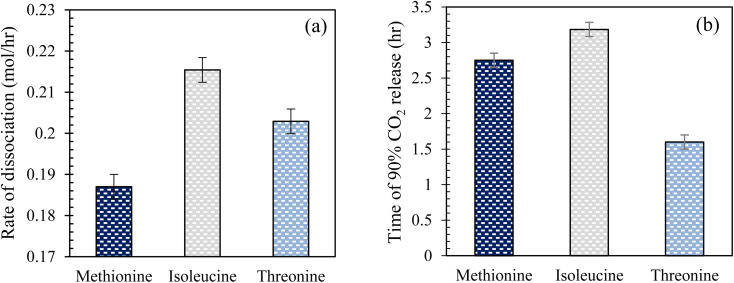
(a) Rate of CO_2_ hydrate dissociation and (b) time taken to release 90% CO_2_ gas at 0.2 wt% l-meth, l-iso, and l-threo in quartz sand with brine at 277.15 K.

Fig. 10 illustrates the rate of CO_2_ hydrate dissociation and the time of 90% CO_2_ release in the presence of 0.2 wt% l-meth, l-iso, and l-threo solutions in brine in quartz sand. The time of CO_2_ release and hydrate dissociation rate are the key indicators that determine CO_2_ hydrate stability in quartz sand. The rate of dissociation gives a measure of how fast or slow the formed hydrates would dissociate and release the gas trapped within the hydrate. However, the time for CO_2_ release describes the time taken for the gas to be released resulting from the hydrate dissociation process. For CO_2_ sequestration applications in porous sediments, a lower dissociation rate with a prolonged time of CO_2_ release is preferred to delay the rate of release of CO_2_ gas, leading to higher stability of the hydrates. As observed from Fig. 10(a), l-meth exhibits the lowest hydrate dissociation rate followed by l-threo, indicating enhanced stability of the formed CO_2_ hydrates in quartz sand. However, l-iso shows the highest dissociation rate, indicating the lowest hydrate stability in quartz sand. The hydrate dissociation rate in l-meth is lower by 15% and 8.5% as compared to l-iso and l-threo systems, respectively. This indicates that l-meth provides greater hydrate dissociation stability as compared to l-iso and l-threo systems. On the other hand, l-iso shows the strongest kinetic inhibition with increased hydrate dissociation rate among the studied systems in quartz sand. However, the time observed for 90% CO_2_ release is reduced by 15% in the case of l-meth as compared to l-iso (Fig. 10(b)), which indicates that the formed hydrates are mildly stable in l-meth as opposed to the l-iso system. The high hydrate dissociation rate coupled with prolonged release of CO_2_ gas indicates moderate stability of hydrates in the case of l-iso in quartz sand. Nevertheless, the high hydrate dissociation rate of l-iso makes it unsuitable for CO_2_ sequestration applications as hydrates in porous sediments. Further, the time of CO_2_ release and hydrate dissociation rate is lower by 42% and higher by 8.5% for l-threo as opposed to the l-meth system in quartz sand, respectively. This confirms the inhibition effect of l-threo on CO_2_ hydrate dissociation kinetics in quartz sand, suggesting it is unfit for CO_2_ sequestration applications. However, it could serve as an excellent inhibitor for hydrate mitigation applications. This means the formed hydrates in the l-threo system are highly unstable and would dissociate quickly, releasing the trapped CO_2_ as opposed to the l-meth system.

Comparatively, l-meth exhibits better performance by moderately lowering the CO_2_ hydrate dissociation rate, and on average, prolonging the time of CO_2_ release in quartz sand (Fig. 10(b)). This indicates a mild promotion effect of l-meth on CO_2_ hydrate dissociation kinetics in quartz sand. Thus, it could be used for CO_2_ storage applications in porous media containing brine. In the next section, the performance of l-meth is compared with commercial promoter SDS and base brine system to evaluate the effect of each on CO_2_ hydrate dissociation kinetics in quartz sand.

#### Comparison of CO_2_ hydrate dissociation rate and time of CO_2_ release using l-meth, SDS and brine in quartz sand system

**Fig. 11:**
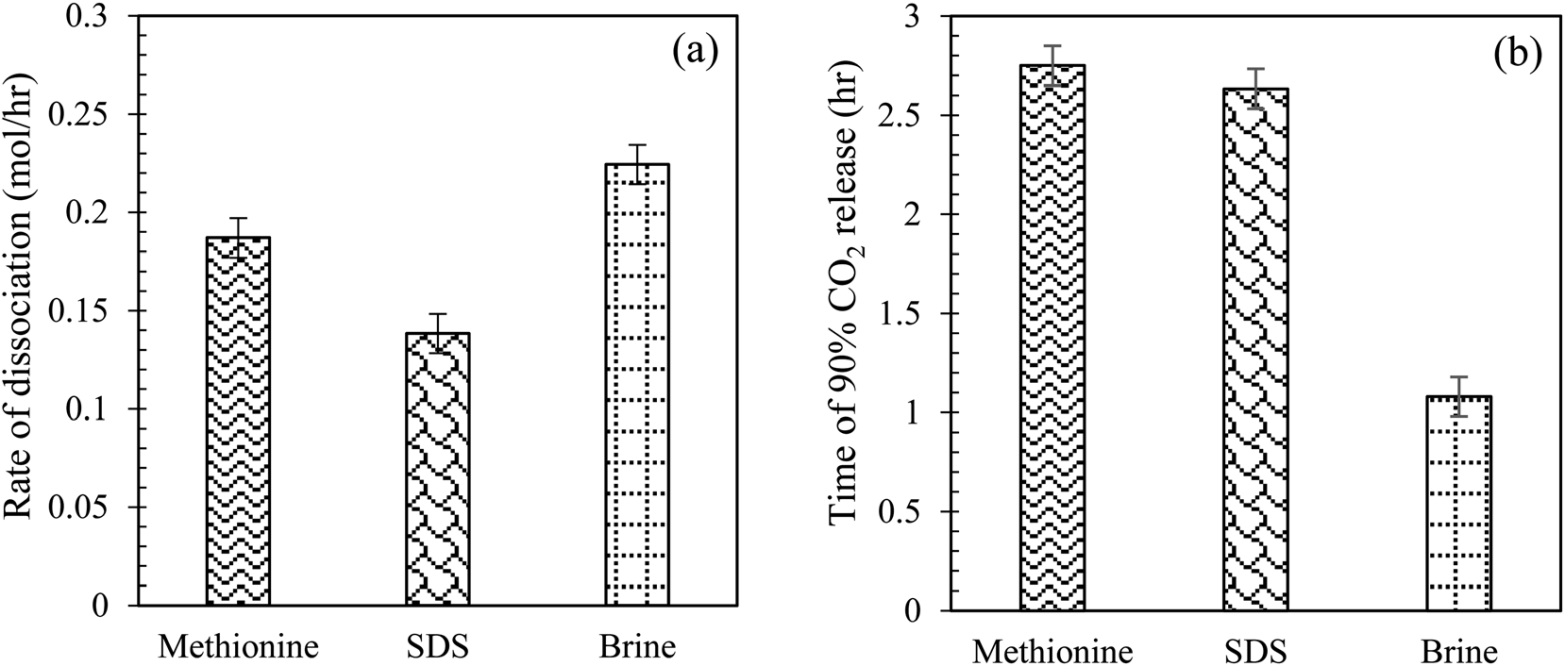
(a) CO_2_ hydrate dissociation rate (mol h^−1^); (b) time taken to release 90% of CO_2_ gas (h) at 0.2 wt% of l-meth, SDS, and 3.3 wt% brine system in quartz sand at 277.15 K.

Herein, the effect of l-meth on CO_2_ hydrate dissociation kinetics is compared with SDS at 0.2 wt% and base brine system by estimating the hydrate dissociation rate and time of 90% CO_2_ release in quartz sand (Table 3). The brine system shows the highest CO_2_ hydrate dissociation rate followed by l-meth, while SDS shows the lowest hydrate dissociation rate (Fig. 11(a)), indicating higher stability of the formed hydrates, as opposed to l-meth in quartz sand. The rate of hydrate dissociation is lower in SDS by 35% as opposed to l-meth in quartz sand. Thus, SDS exhibits a kinetic promotion effect over the l-meth system by stabilizing the CO_2_ hydrates in quartz sand. This indicates that in the presence of brine, SDS shows enhanced performance than l-meth, providing higher hydrate stability in quartz sand. Further comparing brine (base system) with SDS, it shows a significant reduction (38%) in the hydrate dissociation rate, resulting in increased stability of formed CO_2_ hydrates in quartz sand. On the other hand, the time observed for 90% of CO_2_ gas released is the lowest for brine (base system) while prolonged in both l-meth and SDS systems in quartz sand (Fig. 11(b)). The difference in the time of CO_2_ release between l-meth and SDS is almost negligible, as observed in Fig. 11(b). This indicates that both l-meth and SDS exhibit a kinetic promotion effect as opposed to brine (base system) by delaying the release of CO_2_ gas. However, considering the rate of CO_2_ hydrate dissociation, SDS shows enhanced performance than l-meth in quartz sand. The promotion effect of SDS could result from surface activity, as it is known to reduce the surface tension between the gas–liquid contact. Thus, compared to commercial promoter SDS, l-meth fails to provide efficient hydrate stability in terms of the rate of CO_2_ hydrate dissociation.

## Conclusions

This study evaluates the influence of amino acid solutions in a brine system on CO_2_ hydrate formation and dissociation kinetics in quartz sand at 4 MPa, 274.15 K and 277.15 K, respectively. Amongst the studied amino acids, l-meth performed the best with the maximum CO_2_ storage capacity recording about 93% CO_2_-to-hydrate conversion and exhibited the lowest hydrate dissociation rate compared to l-iso and l-threo systems in quartz sand with brine. Further, comparing the results with SDS and brine systems, l-meth enhanced CO_2_ uptake by 36% and 59% in quartz sand, indicating a kinetic promotion effect. The promotion effect of the l-meth system could be because of favorable alkyl chain length as well as increased solubility of l-meth in the brine system, causing a salting-in effect. This leads to better interaction of l-meth with the surface hydroxyl groups on quartz sand *via* covalent bonding, thus exhibiting an enhanced hydrate promotion effect compared to l-iso and l-threo systems in brine. Furthermore, l-meth showed higher hydrate stability with the lowest CO_2_ hydrate dissociation rate compared to l-iso and l-threo systems. However, compared with SDS and base brine, l-meth exhibited slightly higher and considerably lower hydrate dissociation rates, respectively. The classical nucleation theory (CNT) accurately predicted the hydrate nucleation time of CO_2_ hydrate formation in quartz sand with and without the best-studied amino acid l-meth with an average absolute deviation of 2.4 hours. The findings in this study provide insightful knowledge on the use of amino acids as promoters for CO_2_ sequestration as hydrates in sediments. Aside from the measured indicative observations on hydrate formation and stability in this work, we further recommend the use of real-time NMR, Raman, and FTIR characterization techniques to provide a deep molecular-level behavioral understanding of CO_2_ hydrate storage phenomena, hydrate morphology and saturation in sediments in the presence of amino acids.

## Author contributions

BL and MYK supervised the project. ANR, BL, and CBB conceptualized the work and designed the experiments. ANR and CBB carried out the hydrate kinetics and dissociation experiments. ANR and CBB carried out the results analysis and data presentation. ANR wrote the original manuscript draft. ANR, CBB, MYK, and BL edited the manuscript and contributed to the final version. All authors read the final manuscript and consented to the submission.

## Conflicts of interest

There are no conflicts to declare.

## Supplementary Material

RA-014-D4RA00330F-s001
