# Supplementary material for: Amino acid-assisted effect on hydrate-based CO2 storage in porous media with brine
Source: RSC Adv. 2024 Mar 19;14(13):9339–50. doi: 10.1039/d4ra00330f (PMC10950056; doi:10.1039/d4ra00330f)
Supplement: RA-014-D4RA00330F-s001 [file RA-014-D4RA00330F-s001.pdf]

Electronic Supplementary Material (ESI) for RSC Advances.
This journal is © The Royal Society of Chemistry 2024

Supporting Information

Amino Acids assisted effect on hydrate-based CO_2 Storage in Porous Media with Brine

Amirun Nissa Rehman[a,*], Cornelius B. Bavoh[b,c], Mohd Yusuf Khan[a], Bhajan Lal[b,*]

[a] *Interdisciplinary Research Center for Hydrogen Technologies and Carbon Management, King Fahd University of Petroleum & Minerals, Dhahran, 31261 Saudi Arabia.*

[b] *Chemical Engineering Department, Universiti Teknologi PETRONAS, Bandar Seri Iskandar, 32610 Perak Darul Ridzuan, MALAYSIA*

[c] *Department of Chemical and Petrochemical Engineering, University of Mines and Technology, P.O. Box 237, Tarkwa, Ghana*

*Corresponding author: Email: bhajan.lal@utp.edu.my; amirunrehman2728@gmail.com
Phone: (O) +6053687619; (HP) +60103858473; Fax: +6053656176

Number of pages: 5

Number of tables: 2

Number of figures: 3

- Table S1. Properties of the unconsolidated quartz sand.

Table S1. Properties of the unconsolidated quartz sand.

Porous media	Particle size (mm)	Average pore size (nm)	Porosity (%)	Surface area (m^2/g)
Quartz sand	0.6 - 0.8	8.13420	~38	1.1000

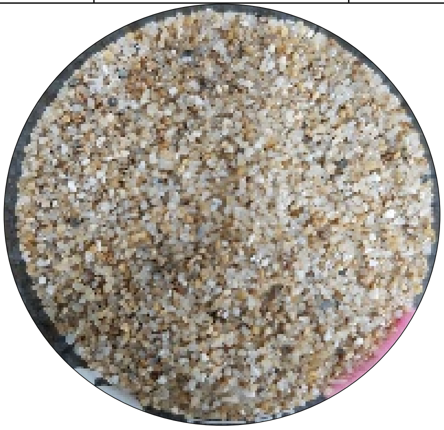

Figure S1. Image of the sorted unconsolidated quartz sand.

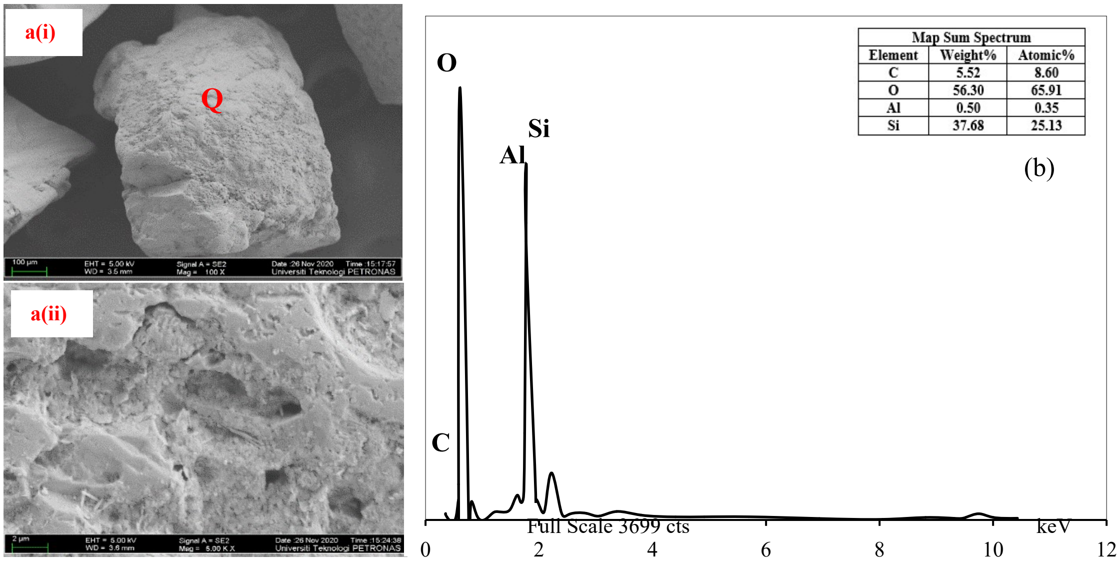

Figure S2. (a) FESEM images of unconsolidated sand showing crystalline quartz grains; (b) EDX spectrum of the unconsolidated sand showing predominant silica (Si) composition

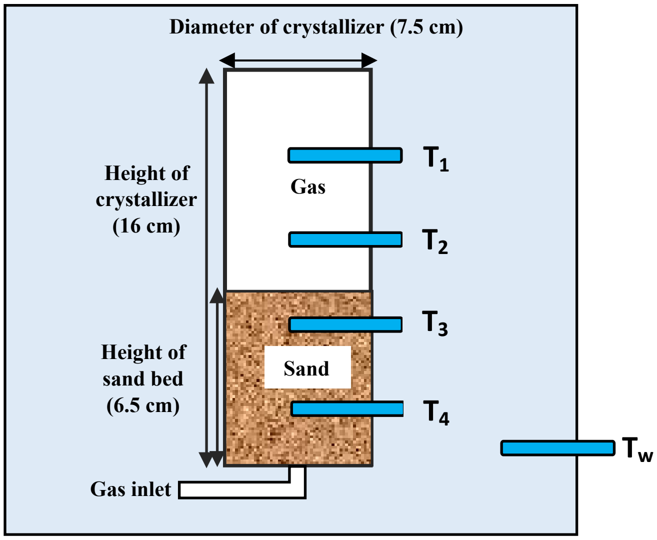

Figure S3. Schematic diagram of the inner cell of sandstone hydrate reactor.

Table S2. Repeated runs of measured CO_2 hydrate formation kinetic data at 0.2 wt.% of L-meth, L-iso, L-threo and SDS in 3.3 wt.% brine solution at 4 MPa and 274.15 K in quartz sand.

System	Induction time (hr)			CO_2 gas consumed (mol)			CO_2 gas uptake (mol/mmol)			Hydrate formation rate (mmol/hr)			C*gh* (%)			C*wh* (%)		
	Run 1	Run 2	Mean	Run 1	Run 2	Mean	Run 1	Run 2	Mean	Run 1	Run 2	Mean	Run 1	Run 2	Mean	Run 1	Run 2	Mean
Brine	31	25	28	0.3970	0.4570	0.4270	53.10	61.12	57.11	7.174	7.814	7.494	31.86	36.67	34.27	35.04	40.34	37.69
0.2-Methionine	89	111	100	0.6968	0.6168	0.6568	96.43	85.36	90.89	1.773	2.573	2.173	57.28	50.70	53.99	63.01	55.77	59.39
0.2-Isoleucine	120	104	112	0.2833	0.2413	0.2623	39.21	33.39	36.30	0.136	0.616	0.376	23.29	19.84	21.56	25.62	21.82	23.72
0.2 Threonine	14.6	18.6	16.6	0.4336	0.4838	0.4587	53.33	59.50	56.41	4.927	5.927	5.427	32.00	35.70	33.85	35.19	39.27	37.23
0.2 SDS	165	135	150	0.5759	0.4939	0.5349	71.81	61.58	66.69	1.242	1.602	1.422	43.08	36.95	40.02	47.39	40.64	44.02
